# What are developers talking about information security? A large-scale study using semantic analysis of Q&A posts

**DOI:** 10.7717/peerj-cs.1954

**Published:** 2024-03-26

**Authors:** Fatih Gurcan

**Affiliations:** Department of Management Information Systems, Faculty of Economics and Administrative Sciences, Karadeniz Technical University, Trabzon, Turkey

**Keywords:** Information security, Q&A communities, Empirical study, Topic modeling, Semantic analysis

## Abstract

**Background:**

Digitalization and rapid technological improvement in the present day bring numerous benefits, but they also raise the complexity and diversity of cyber security risks, putting critical information security issues on the agenda. Growing issues and worries about information security endanger not only the security of individuals and organizations but also global social and economic stability.

**Methods:**

This study investigates the issues and challenges regarding information security by analyzing all the postings on ISSE (Information Security Stack Exchange), a Q&A website focused on information security. In order to identify the primary topics addressed in postings shared on the ISSE platform, we employed a probabilistic topic modeling method called latent Dirichlet allocation (LDA), which is generative in nature and relies on unsupervised machine learning processes.

**Results:**

Through this investigation, a total of 38 topics were identified, demonstrating the present state of information security issues and challenges. Considering these topics, a comprehensive taxonomy of seven categories was devised to address information security issues, taking into account their backgrounds and perspectives. Subsequently, we conducted an examination of the prevalence and complexity of the matters at hand. In addition, we have defined the prevailing technologies utilized in the realm of information security, including tasks, certifications, standards, methods, tools, threats, and defenses. We have provided a number of implications for different stakeholders, including academics, developers, educators, and practitioners, who are working towards advancing the field of information security.

## Introduction

In the era of digital transformation, sometimes referred to as the “Information Age”, there has been a tremendous advancement in the production and sharing of information. This has resulted in a significant rise in both the quantity and variety of information available, with improved access and sharing capabilities. The services and applications provided in the digital age have exposed the need to address more complex information security concerns. The proliferation of the Internet, advancements in cloud computing technologies, the rise of online shopping and marketing, the availability of online finance and banking services, the popularity of social networks, and the widespread use of mobile applications have expanded the accessibility of information for individuals and institutions (McCormac et al., 2017; Edu, Agozie & Agoyi, 2021; Whitman & Mattord, 2021; Gurcan et al., 2023). However, these developments have also introduced new vulnerabilities and risks to information security (Glaspie & Karwowski, 2018; Whitman & Mattord, 2021). In general terms, the privacy and security of information shared through online services and applications that users commonly use is an important issue that closely concerns all online user societies (McCormac et al., 2017). Applications that record the private information of users and institutions have led to an increase in developer activities around information security, and thus it has become a challenging and strategic work area for today’s IT professionals (Glaspie & Karwowski, 2018; Whitman & Mattord, 2021). In general, the privacy and security of personal information shared through widely used online services and applications is an important issue that closely concerns everyone (Soomro, Shah & Ahmed, 2016; McCormac et al., 2017). Applications that record the private information of users and institutions have led to an increase in developer activities around information security, and thus it has become a challenging and strategic work area for today’s IT professionals (Furnell, Fischer & Finch, 2017; Glaspie & Karwowski, 2018).

Information security is a growing and evolving field, covering a wide range of areas, from network and infrastructure security to testing and auditing, from risk management to security policies (Whitman & Mattord, 2021). Information security includes a set of security procedures and tools that broadly protect confidential corporate and personal information from misuse, unauthorized access, corruption, or destruction (Soomro, Shah & Ahmed, 2016; Yang et al., 2016). Information security covers physical and perimeter security, access control, and cybersecurity. Information security protects sensitive information from unauthorized activities, including inspection, modification, recording, and any interruption or destruction (Whitman & Mattord, 2021). The purpose is to ensure the security and privacy of critical data such as customer account details, financial data, or intellectual property (Soomro, Shah & Ahmed, 2016; McCormac et al., 2017). The proliferation of services and applications where information security is essential brings with it many challenges for their development. In other words, digital transformation based on information technologies has increased the responsibilities of information security experts (Furnell, Fischer & Finch, 2017; Glaspie & Karwowski, 2018; Gurcan et al., 2023).

Presently, services and applications that prioritize information security encompass a diverse range of jobs, technologies, techniques, and paradigms. Proficiency in a diverse variety of knowledge domains and skill sets is necessary for information security professionals (Yang et al., 2016). Even seasoned information security experts may struggle to stay abreast of the swift advancements in these frameworks and technologies (Yang et al., 2016; Furnell, Fischer & Finch, 2017; Glaspie & Karwowski, 2018).

Because of these factors, information security professionals frequently turn to domain-specific Q&A platforms, like the Information Security Stack Exchange (ISSE), to get guidance and support from their peers regarding the technical problems and obstacles they encounter (Stack Exchange, 2023). ISSE seeks answers to information security-oriented problems from field professionals from various backgrounds by controlling the postings posted by thousands of users (Stack Exchange, 2023). Due to the advancements in information security-related activities in the past few years, the ISSE platform has emerged as a crucial repository of knowledge and a valuable point of reference for professionals in the information security industry. All shares on ISSE can be considered a valuable data repository that keeps track of the challenges, focuses, and experiences of information security authorities from the past to the present (Stack Exchange, 2023). Analyzing this pool can provide important inferences about the themes and backgrounds of the technical problems and difficulties encountered, which tools, paradigms, and technologies are preferred for information security, and what is needed to solve security problems (Barua, Thomas & Hassan, 2014; Yang et al., 2016).

From this perspective, numerous academics have recently conducted research using data from Stack Overflow to explore certain aspects of software design and development, including chatbot development, security, testing, mobile development, and IOT development (Vasilescu, 2014; Ahmad et al., 2018). Although there have been some valuable initiatives, there is currently a lack of comprehensive studies that investigate the entire field of information security utilizing topic modeling-based semantic content analysis of data from online Q&A platforms (Vasilescu, 2014), with only a few notable exceptions (Yang et al., 2016; Lopez et al., 2018, 2019; Croft et al., 2022). Undoubtedly, we anticipate that the analysis of data from interactive Q&A communities will have noteworthy inferences and insights for comprehending the chronological progression of information security.

Taking into consideration the aforementioned context, the objective of this study is to conduct a comprehensive analysis of prevalent issues and difficulties associated with information security, thereby addressing a gap in the existing body of knowledge. For this purpose, an experimental *corpus* containing all the posts shared in the last 12 years from November 2010 to October 2022 on ISSE, an information security-focused Q&A platform, was analyzed with a probabilistic issue modeling approach based on unsupervised machine learning. As a consequence of the investigation, we identified the fundamental challenges and themes in information security and, at the same time, the indications, underlying relationships, and long-standing tendencies. In brief, we provide the rationale and outline the research problems that will be addressed in our experimental analysis.

**RQ1.** What information security issues are addressed?

**RQ2.** How do information security issues evolve over time?

**RQ3.** How have the difficulty and popularity of information security issues progressed over time?

**RQ4.** In the field of information security, which tasks, techniques, and tools are the most frequently employed?

### RELATED WORK

Information security existed prior to the development of the computer. According to Rusell & Gangemi (1991), information security has been around as long as information (Rusell & Gangemi, 1991). Information has required security since it was first sent, stored, and processed. This dates back to when people first started to write. Denning (1999) transports us back to the first century, when Julius Caesar created a code of secrecy to avoid the interception of secret communications transmitted to his friends (Denning, 1999). From the earliest days of communication, diplomats and military commanders understood that it was necessary to provide some mechanism to protect the confidentiality of correspondence and to have some tools to detect security breaches, and they took initiatives to ensure information security. Later, procedural transaction control processes and practices were mostly used to ensure information protection. With the spread of postal services, governments created official bodies to seize, decipher, read, and reseal letters (for example, the United Kingdom Secret Office, established in 1653) (Johnson, 1998). At the end of the 20th century and in the first years of the 21st century, telecommunications, computer hardware and software, and data encryption all changed quickly and in new ways. Innovations and new ideas in the 21st century have made it clear how important IT infrastructure is for information security (Yang et al., 2016). Companies have realized that their ability to stay competitive in highly volatile and changing markets depends on how well they protect their information assets and IT infrastructures from security risks (Soomro, Shah & Ahmed, 2016; Ključnikov, Mura & Sklenár, 2019). The fact that all of these new technological advancements are susceptible to hazards and negative effects, just like any other new digital inventions, is now widely acknowledged by the IT communities (McCormac et al., 2017).

In line with the developments since its existence, information security has become a common concern and work area not only of the IT communities but also of most industries and academies (Silic & Back, 2014; Glaspie & Karwowski, 2018). Numerous workshops and conferences were held to form joint working groups for information security, discuss information security issues, and improve cooperation (Dlamini, Eloff & Eloff, 2009; Silic & Back, 2014; Soomro, Shah & Ahmed, 2016). With these interactions and activities, the continuous participation of the ever-expanding communities in the field has increased the popularity of information security day by day. While the information security paradigm has developed rapidly, the number of studies in the information security literature has increased rapidly (Whitman & Mattord, 2021).

Numerous literature reviews have been conducted so far to understand the information security paradigm (Dlamini, Eloff & Eloff, 2009; Silic & Back, 2014; Soomro, Shah & Ahmed, 2016; Glaspie & Karwowski, 2018). Domain-specific studies have generally addressed information security architectures, techniques, and tools (Whitman & Mattord, 2021), risk analysis and management (Soomro, Shah & Ahmed, 2016), threats to information security and solutions (Lopez et al., 2018), applications in different fields (Silic & Back, 2014), information security awareness (McCormac et al., 2017; Albayrak & Bağcı, 2022), the development of secure protocols (Dlamini, Eloff & Eloff, 2009), and the evolution and future of information security (de Leeuw & Bergstra, 2007; Stewart, 2013). These reviews offered insights into the themes and trends of information security in both academia and industry (Furnell, Fischer & Finch, 2017; Glaspie & Karwowski, 2018).

In addition, numerous studies have focused on online Q&A forums similar to Stack Overflow (Stack Exchange, Quora, Kaggle, and Reddit), which is a crucial venue for developers from diverse backgrounds in the IT industry to share knowledge and experiences (Vasilescu, 2014; Ahmad et al., 2018; Croft et al., 2022). In particular, several studies have been carried out based on the examination of posts from Q&A forums to investigate the issues and difficulties faced by developers and security specialists in various cybersecurity scenarios (Yang et al., 2016; Lopez et al., 2018, 2019; Croft et al., 2022). Yang et al. (2016) analyzed the security-related posts of developers on the Stack Overflow Q&A website with a topic modeling approach. As a result of this analysis, they classified safety-related topics into five main categories (Yang et al., 2016). Lopez et al. (2019) created an anatomy of security conversations using a manual categorization on Stack Overflow. From a similar perspective, Lopez et al. (2018) analyzed a series of questions in the security channel of Stack Overflow to draw inferences about how developers use Q&A resources to solve security problems. Croft et al. (2022) conducted a large-scale study of the security challenges of 15 programming languages by analyzing developers’ discussions on Stack Overflow and GitHub using a topic modeling approach.

The scope of our study encompasses novel methodologies and a more detailed dataset, which builds upon and expands earlier research. Our study analyzes all posts shared on ISSE using a semi-automated methodology that is not influenced by author prejudice. We employ a topic modeling approach based on unsupervised machine learning to address this gap in the existing literature. Topic modeling generates the word clusters that best define the semantic map of the contents as different themes (Blei, 2012; Gurcan & Cagiltay, 2022). Owing to the methodological advantages it offers over semantic content analysis, topic modeling is growing in popularity for unstructured textual data (Gurcan et al., 2022b; Ozyurt et al., 2022). Therefore, numerous studies have been conducted using topic modeling procedures in sub-contexts of various disciplines (Chen, Thomas & Hassan, 2016; Silva, Galster & Gilson, 2021). In conclusion, topic modeling has proven to be an effective and appropriate way for semantic analysis and interpretation of unstructured texts, which has further motivated us to use this methodology to investigate information security challenges.

## Method

### Data query and retrieval

To make available a systematic and unbiased methodological approach, we investigated an archive of all postings generated on the Information Security Stack Exchange (ISSE), an information security-focused Q&A community. (Stack Exchange, 2023). The XML data dump of the datasets generated and examined for the present study is accessible to the general public through the Internet Archive repository (Internet Archive, 2023). Initially, the up-to-date XML data dump (updated October 8, 2022) was downloaded and parsed into a relational data file before being processed. There are two different types of posts in the data dump: question and answer posts. In total, the experimental *corpus* includes 180,937 posts published from November 2010 to October 2022, of which 66,077 are questions and 114,860 are answer posts. In addition, it has been determined that approximately 5,082 questions and 8,835 answers are shared on the ISSE platform on average every year. Each question post has a number of metadata components, including title, tags, body, code, answers, comments, and further information. (Yang et al., 2016). Answer posts, unlike question posts, do not have title or tag elements.

### Data preprocessing

We perform a five-step data preprocessing to transform the unstructured textual raw data in the experimental *corpus* into noise-free and structured data. Since posts on Stack Overflow often contain code snippets, we removed them in the first step (Uddin et al., 2021). Since code snippets in posts on Stack Overflow often contain syntax and code specific to programming languages, they have no semantic contribution to topic modeling and can degrade the performance of the analysis (Barua, Thomas & Hassan, 2014). Also, because the shared source code on Stack Overflow contains only small pieces of code, there is not enough semantic context to derive meaningful topic models from code snippets. Therefore, we clean up the code snippets marked with <code></code> from posts using a similar approach to previous work. Since HTML tags (*e.g*., <p>, <pre>, and <b>) and URLs do not contribute to the creation of nautical topics in the topic modeling process, we remove all HTML tags and URLs from the text in the *corpus* (Croft et al., 2022). In step three, we continue to remove numbers, punctuation, and other non-alphabetic characters from the dataset to enable better topic modeling results. As a fourth step, we deleted all English stop words (how, what, and, is, or, the, a, an, for, *etc*.) that don’t make sense on their own (Gurcan et al., 2022a). Finally, we applied the lemmatization process to the remaining words in order to reduce the words from their derived form to their nominative form (Gurcan & Cagiltay, 2022).

### Topic modeling analysis

Our goal is to uncover debate topics related to distinct contexts of information security in our dataset from the ISSE platform. In order to address this issue, we employ a topic model utilizing the latent Dirichlet allocation (LDA) algorithm to investigate and establish specific debate topics within various information security frameworks. LDA is a statistical and generative approach for topic modeling, where topics are represented as probability distributions over words in a collection of texts (Blei, Ng & Jordan, 2003). Additionally, it provides a concise overview of the documentation by representing the detected topics as probability distributions. LDA employs word frequencies and word co-occurrences across documents to construct a semantic topic model consisting of interconnected terms (Gurcan, Dalveren & Derawi, 2022; Gurcan et al., 2023). Words pertaining to a given issue are typically connected in meaning, thereby attributing a distinct significance to the topic in consideration. Conversely, because of the absence of semantic information in the LDA model, the meanings of the linked topics must be ascertained through manual examination of the word set. The presence of phrases such as “encrypt”, “data”, “encryption”, “decrypt”, “unencrypted”, and “plaintext” in a topic suggests that the issue is likely to be connected to encryption in the field of information security (Blei, 2012). LDA has gained widespread adoption as an approach for extracting semantic themes from unstructured textual materials. The topics generated by LDA are less prone to overfitting and are more easily interpretable (Vayansky & Kumar, 2020). Hence, a substantial body of research has surfaced in the domain of software engineering employing topic modeling techniques (Chen, Thomas & Hassan, 2016; Silva, Galster & Gilson, 2021).

We utilize the LDA model implementation offered by the Gensim package to employ LDA on our experimental *corpus* (Řehůřek & Sojka, 2011). For every topic modeling experiment based on LDA, we increment the number of topics, designated as K, one by one while keeping all other parameters at their default values. The K parameter, denoting the quantity of topics, is a variable typically chosen by the user and determines the level of detail of the topics (Gurcan, Dalveren & Derawi, 2022). Having a K number that is neither too large nor excessively small is considered unsuitable. Excessive K values might result in topic relocation and repetition, while insufficient K values may lead to the identification of broader, partially differentiated topics. We conducted a series of experiments using various K values in order to identify themes that exhibited the greatest level of consistency (Vayansky & Kumar, 2020). In this context, coherence scores are employed to evaluate the significance and coherence of the topics that have been identified. In this empirical process, we systematically varied the K range from 5 to 60, increasing by 1 at each iteration (*e.g*., 5, 6, 7,…, 60). In this way, we identified the optimal K value that generates topics with high coherence scores (Croft et al., 2022). Concurrently, we computed a coherence score that is tailored to the LDA model for each experiment that is fitted with the K value. We used the four-step topic coherence pipeline and the coherence model built into the Gensim module to do this computation (Mimno et al., 2011; Řehůřek & Sojka, 2011; Röder, Both & Hinneburg, 2015; Gurcan, 2023a). A high coherence score signifies that the themes exhibit greater distinctiveness from one another in the semantic domain. Ultimately, we selected the topic model that had the highest coherence score. The study revealed that the coherence score for K = 38 achieved its highest value (Cv = 0.7189). Hence, after analyzing the findings, we determined that 38 is the most suitable number of topics.

Applying LDA-based topic modeling to our *corpus* using the sequential processes mentioned above resulted in the generation of 38 topics. The chosen model provides the following metrics and information for each identified topic: (1) Keywords that provide a detailed description: A ranked list of the N most frequently occurring words that describe the topic, along with the probability of each word. This probability indicates the relative dominance of each word in terms of its descriptive power for the topic. We discovered the 20 most prominent words for each topic. (2) Distribution of the topics: Every post is assigned a correlation value ranging from 0 to 1, indicating the degree of association between each post and the themes. A post is deemed more “on-topic” when it has a higher correlation to a specific topic. We allocated a post to a topic based on the highest correlation values between the post and the topic (Uddin et al., 2021; Gurcan, 2023b).

After the postings were allocated to the prevailing topics, we carried out the task of determining topic tags that briefly summarize the conceptual background of each topic. We manually assigned tags to each topic using an open-card sorting approach, taking into account previous research on topic labeling (Katsanos et al., 2019). In open-card sorting, there is no previously determined label for a topic. Instead, this label is determined using the open coding process (Katsanos et al., 2019; Uddin et al., 2021). To classify a topic, we utilize two forms of information: (1) a compilation of the most frequently employed words in the topic, and (2) a selection of 20–30 articles on that topic chosen at random. Based on this information, the label for each topic was derived and assigned. During the card sorting process, the coders, who are the experts in the subject, assigned a label to each topic. Finally, the 38 topics were classified using the open-card sorting method, considering their background (Katsanos et al., 2019; Croft et al., 2022). As a result, a comprehensive taxonomy map was developed to categorize and map the primary themes, contexts, and application areas within the field of information security. The map is organized into seven categories.

## Results

Within this section, we shall provide the outcomes of our analysis in the form of answers to each research question, organized into four subheadings.

### What information security issues are addressed? (RQ1)

Our analysis employed LDA-based topic modeling to examine the information security concerns raised on the ISSE platform. The method section provides a comprehensive explanation of our modifications and implementation of LDA-based topic modeling in our *corpus*. Through the utilization of LDA-based semantic content analysis, a total of 38 topics were identified, with each topic being characterized by 20 descriptive keywords. Table 1 presents the 38 topics (issues) identified by LDA-based topic modeling, along with their corresponding percentages. The themes (problems) are arranged in Table 1 in descending order based on their respective percentages. Furthermore, Table A1 provides the top 20 keywords associated with each topic. The words “topic” and “issue” are utilized interchangeably in the current study due to the fact that the topics listed in Table 1 represent significant issues and challenges related to information security. Topics showed that information security issues cover a wide range of topics, from “Cyber Attacks” to “XSS Attacks”, “Security Testing” to “Code Vulnerability”, “Certification” to “Encryption” (see Table 1).

**Table 1:** The 38 topics discovered by LDA.

No	Topic name	Rate (%)	No	Topic name	Rate (%)
1	Cyber attacks	5.75	20	Entropy	2.38
2	Security testing	3.93	21	HTTP proxy	2.37
3	Certification	3.90	22	E-Mail	2.23
4	User account	3.82	23	Buffer overflow	2.19
5	Wi-Fi networks	3.69	24	Authentication	2.16
6	XSS attacks	3.66	25	VPN	2.15
7	Corporate data	3.65	26	Encryption/Decryption	2.14
8	Logging	3.61	27	File transmission	2.09
9	Website	3.40	28	Mobile apps	2.02
10	Port scanning	3.14	29	Cipher suites	2.02
11	SSH access	3.00	30	Phone scam	1.90
12	Encryption keys	2.96	31	Block cipher	1.88
13	Malware	2.95	32	DNS	1.86
14	Access control	2.74	33	Data backup	1.79
15	Password hashing	2.70	34	Credit card	1.77
16	Web API	2.68	35	Disk encryption	1.65
17	TLS connection	2.63	36	Virtual machine	1.53
18	CSRF	2.53	37	SQL injection	1.47
19	Code vulnerability	2.42	38	Digital signature	1.22

Moreover, the five most commonly asked topics across the board were “Cyber Attacks”, “Security Testing”, “Certification”, “User Account”, and “Wi-Fi Networks”. The least asked topics were “Virtual Machine”, “SQL Injection”, and “Digital Signature”. Especially in the information technology ecosystem, where even highly secure systems are exposed to cyber-attacks, preventing these attacks and making the systems protected against them has emerged as the main problem focused on by information security experts. The fact that “Cyber Attacks” and “Security Testing” are at the top of the list is a finding that confirms this. The topics discovered also shed light on the priorities and current trends in the ever-growing information security industry. Information security topics cover a wide range of knowledge, skills, and backgrounds in different areas of expertise. The broad spectrum of information security is clearly reflected in the scope of the topics explored.

**Figure 1:**
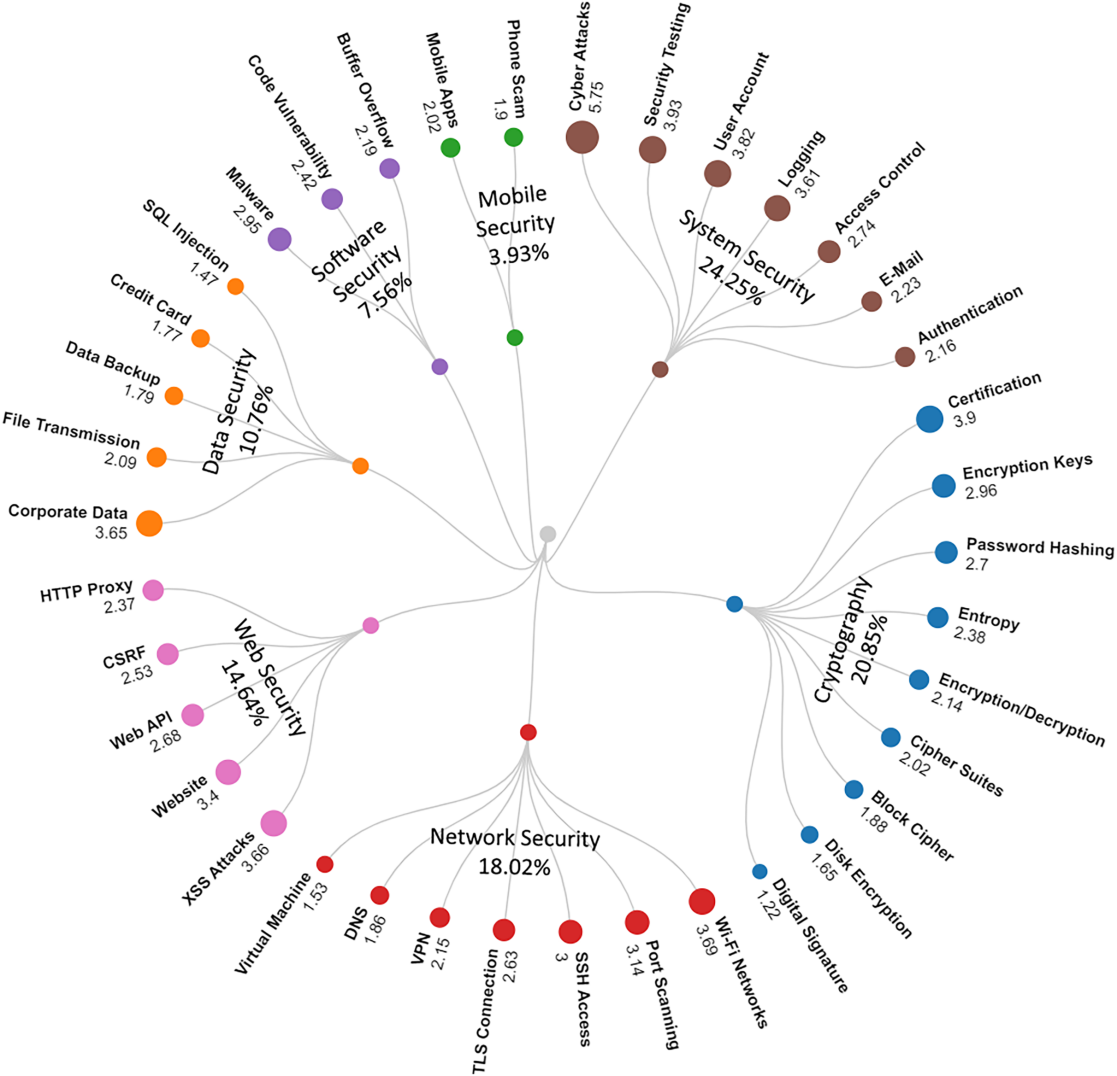
Taxonomy of the topics.

In order to make the main themes of information security more understandable, a categorization process was carried out by associating these topics with their conceptual background and processes. The 38 information security topics were classified under seven elementary categories, and a structural taxonomy map was developed for information security and presented in Fig. 1. As shown in Fig. 1, information security issues are classified under seven categories: “System Security” (24.25%), “Cryptography” (20.85%), “Network Security” (18.02%), “Web Security” (14.64%), “Data Security” (10.76%), “Software Security” (7.56%), and “Mobile Security” (3.93%). Issues in the “System Security” (24.25%) and “Cryptography” (20.85%) categories account for almost half of the information security issues. This indicates that “System Security” and “Cryptography” categories dominate among information security issues. On the other hand, it was seen that the issues in the “Software Security” (7.56%) and “Mobile Security” (3.93%) categories had relatively the lowest percentages.

### How do information security issues evolve over time? (RQ2)

In order to investigate this research question, we analyze the evolution of issues surrounding information security over a specific timeframe to comprehend the persistent and unresolved issues in information security and generate innovative methods to tackle them. In order to accomplish this, we employed the temporal trend calculating methodology that we utilized in our prior study to disclose the movements of each topic over the last decade (Gurcan, 2023a). Table 2 displays the yearly percentages, trend values, and trend directions for the themes arranged according to the general trend. Each row in this table displays the yearly percentage changes for a topic and provides a number of insights regarding the evolution of the topics over time. Based on the movement values in the final column of Table 2, it can be observed that out of the 38 topics, 28 exhibit an upward trend, while 10 show a downward trend. Topics with the most increasing trend emerged as “Certification”, “Encryption Keys”, “Web API”, “Disk Encryption”, and “VPN”. On the other hand, “Corporate Data”, “Cyber Attacks”, and “Security Testing” are the topics with the most decreasing trend. It is a remarkable finding that although “Cyber Attacks” and “Security Testing” are the two topics with the highest percentage, they have the most noticeable downward trend.

**Table 2:** Temporal trends of the topics.

Topic Name	2010	2011	2012	2013	2014	2015	2016	2017	2018	2019	2020	2021	2022	Trend
Certification	1.55	3.07	3.36	3.31	4.24	4.91	3.85	4.21	3.05	3.67	3.61	4.30	4.79	0.25
Encryption keys	0.62	1.80	2.24	2.52	3.30	3.51	2.85	2.98	3.02	2.78	2.98	3.35	3.53	0.22
Web API	1.24	1.51	2.14	2.58	2.88	2.42	2.25	2.75	2.60	3.26	3.18	3.03	3.89	0.20
Disk encryption	0.62	1.66	1.84	1.38	1.43	1.83	1.50	1.22	1.91	1.38	1.99	2.00	2.82	0.17
VPN	0.62	1.41	1.74	2.21	2.27	2.51	2.16	1.92	2.13	1.76	2.61	2.32	2.55	0.15
Access control	2.17	2.68	2.70	2.44	2.31	2.09	2.86	2.63	3.21	3.01	3.00	3.05	4.07	0.15
Malware	1.86	2.63	3.10	3.15	2.86	2.62	2.81	3.28	2.75	2.82	3.32	2.89	3.76	0.15
E-Mail	0.93	1.27	2.06	1.99	2.10	1.87	2.53	2.16	2.36	2.60	2.31	2.62	2.73	0.14
Phone scam	0.31	0.93	1.15	1.32	1.39	2.09	2.07	2.30	2.03	2.20	2.11	2.38	2.06	0.13
Wi-Fi networks	0.93	3.46	3.15	3.61	3.57	3.66	4.49	4.55	3.27	3.91	3.44	2.67	2.60	0.13
Mobile apps	1.24	1.27	1.20	1.66	1.45	1.77	2.46	2.31	2.21	2.68	1.72	2.49	2.60	0.10
Buffer overflow	0.93	0.97	1.76	2.03	1.87	1.73	2.24	2.52	2.73	2.44	2.59	2.43	2.15	0.09
HTTP proxy	1.55	1.46	1.44	1.44	1.83	2.18	2.48	2.76	2.69	3.04	2.88	3.08	2.68	0.09
Block cipher	0.62	2.68	2.03	3.07	2.32	2.10	1.85	1.33	1.55	1.45	1.44	1.70	1.61	0.08
Logging	3.10	3.26	3.31	3.41	3.57	3.49	3.60	4.17	3.80	3.55	3.59	3.27	3.98	0.07
TLS connection	1.55	2.48	2.32	2.84	3.14	3.08	2.56	2.64	2.01	2.29	2.96	2.40	2.42	0.07
Authentication	2.79	1.61	1.39	1.75	1.80	1.76	2.00	1.76	2.27	2.85	3.10	3.00	3.62	0.06
Data backup	0.93	1.90	1.39	1.69	1.78	1.50	1.99	1.75	1.70	1.78	1.90	2.57	1.70	0.06
Encryption/Decryption	1.24	2.44	1.84	2.19	2.47	2.32	2.12	1.92	2.16	1.78	2.13	2.54	1.97	0.06
Cipher suites	1.24	2.68	1.95	2.58	2.38	2.58	1.86	1.76	1.74	1.84	1.70	1.38	1.97	0.06
Digital signature	0.62	1.17	0.75	1.14	1.26	1.30	1.28	1.18	1.05	1.42	1.24	1.51	1.34	0.06
Password hashing	1.55	3.02	3.79	3.23	3.42	2.89	2.16	2.61	1.98	2.68	2.29	2.70	2.15	0.05
Virtual machine	0.62	1.95	1.17	1.32	1.29	1.15	1.39	1.88	2.58	1.49	1.42	1.51	1.16	0.04
Website	2.48	2.73	3.12	3.63	3.35	3.92	3.36	3.36	3.59	3.38	3.18	3.40	2.73	0.02
File transmission	1.86	1.75	1.66	1.95	1.87	2.01	2.06	2.35	2.27	2.07	2.41	2.27	2.06	0.02
Credit card	0.62	1.66	1.95	1.71	2.24	2.00	1.87	2.04	1.52	1.93	1.36	1.03	0.76	0.01
DNS	2.17	1.22	1.68	1.26	1.26	2.24	2.10	1.88	2.04	1.55	2.49	2.03	2.24	0.01
Entropy	1.86	2.73	2.67	2.64	2.59	2.62	2.42	2.22	2.19	2.14	2.17	2.24	1.83	0.00
CSRF	3.10	1.46	2.03	2.36	2.28	2.14	2.11	2.77	2.57	3.25	3.28	3.22	2.86	−0.02
User account	3.10	3.70	4.01	3.96	4.31	3.82	4.19	3.62	3.80	3.81	3.38	3.46	2.77	−0.02
Port scanning	2.79	3.07	4.62	3.57	3.48	3.00	3.34	2.93	3.26	2.70	2.59	2.54	2.28	−0.04
SQL injection	1.86	1.12	0.96	1.66	1.43	1.51	1.64	1.32	1.65	1.45	1.64	1.38	1.25	−0.05
Code vulnerability	3.41	1.75	2.40	2.15	2.89	2.21	2.66	2.41	2.43	2.05	2.29	2.70	2.68	−0.06
XSS attacks	4.95	2.53	2.96	3.31	3.45	3.08	3.29	3.33	4.59	4.61	4.36	4.35	3.89	−0.08
SSH access	4.95	2.97	3.39	3.29	3.01	2.77	2.85	3.11	2.94	2.66	3.38	2.67	3.31	−0.13
Corporate data	5.57	6.43	4.75	3.88	3.35	3.87	3.70	3.69	3.77	3.33	2.65	2.95	2.15	−0.26
Cyber attacks	11.15	8.77	8.54	7.25	6.25	5.84	5.63	4.84	5.37	4.95	4.54	4.16	4.47	−0.51
Security testing	21.36	10.81	7.42	4.51	3.33	3.64	3.41	3.52	3.30	3.42	2.78	2.40	2.55	−1.45

### How have the difficulty and popularity of information security issues progressed over time? (RQ3)

The results of our investigation in RQ1 unveiled the extensive range and variety of information security issues. The significance, prevalence, and level of complexity of the issues and challenges in information security differ for each specific topic. Certain questions may be presented numerous times, but others may not be presented at all. Assessing the prevalence and complexity of information security issues can aid in the search for effective ways to address these challenges. Various descriptive metrics provide insights into the features of the information security questions shared on ISSE. The aforementioned indicators comprise various parameters, including the number of views, responses, accepted answers, favorites, and comments. During this phase of our analysis, we utilized the metric calculation approach described in our previous study to determine the number of views, answers, accepted answers, favorites, comments, and scores for every topic (Gurcan, 2023a). Subsequently, we computed metrics that ascertain the level of complexity and the level of interest for each topic (Gurcan, 2023a). In order to illustrate all aspects of information security issues, we computed the averages of each topic’s questions, answers, accepted answers, favorites, scores, views, and comments and demonstrated them in Table 3.

**Table 3:** Descriptive indicators of the information security topics.

Topic name	Rate (%)	Question count	View count	Favorite count	Voting score	Comment count	Answer count	Accepted answer count	Difficulty score
Cyber attacks	5.75	3,801	3,606	0.50	8.05	2.71	2.24	0.64	0.50
Security testing	3.93	2,596	2,114	0.48	4.12	2.09	1.86	0.42	0.65
Certification	3.90	2,574	4,100	0.41	5.09	1.68	1.56	0.59	0.61
User account	3.82	2,525	3,478	0.45	8.28	3.02	2.45	0.44	0.58
Wi-Fi networks	3.69	2,439	5,009	0.39	3.55	2.21	1.68	0.42	0.68
XSS attacks	3.66	2,420	3,025	0.38	3.71	2.08	1.53	0.49	0.66
Corporate data	3.65	2,412	3,648	0.45	8.88	3.44	2.33	0.47	0.58
Logging	3.61	2,388	2,865	0.34	3.99	2.74	1.51	0.31	0.75
Website	3.40	2,247	3,841	0.41	5.67	2.56	1.73	0.42	0.67
Port scanning	3.14	2,077	4,922	0.40	2.98	2.09	1.58	0.49	0.66
SSH access	3.00	1,985	2,684	0.34	3.56	1.93	1.65	0.39	0.69
Encryption keys	2.96	1,955	4,001	0.43	5.08	1.80	1.56	0.48	0.66
Malware	2.95	1,949	2,838	0.36	3.46	2.38	1.78	0.41	0.67
Access control	2.74	1,813	3,249	0.39	4.42	1.96	1.58	0.47	0.67
Password hashing	2.70	1,781	5,667	0.48	8.33	2.74	1.99	0.59	0.56
Web API	2.68	1,769	1,568	0.35	2.60	2.14	1.40	0.29	0.78
TLS connection	2.63	1,737	4,594	0.41	5.22	1.97	1.60	0.53	0.63
CSRF	2.53	1,671	4,150	0.40	4.76	1.90	1.58	0.49	0.66
Code vulnerability	2.42	1,600	2,118	0.41	4.86	2.12	1.64	0.43	0.68
Entropy	2.38	1,572	5,139	0.46	8.31	2.79	2.13	0.56	0.56
HTTP proxy	2.37	1,569	3,779	0.38	4.23	2.00	1.52	0.48	0.67
E-Mail	2.23	1,475	3,652	0.37	4.94	2.51	1.82	0.43	0.65
Buffer overflow	2.19	1,445	2,725	0.45	3.49	1.87	1.30	0.44	0.72
Authentication	2.16	1,425	2,889	0.37	4.28	1.50	1.45	0.42	0.71
VPN	2.15	1,421	3,730	0.42	3.83	2.12	1.72	0.45	0.66
Encryption/Decryption	2.14	1,417	2,255	0.38	2.98	2.44	1.64	0.42	0.68
File transmission	2.09	1,378	4,055	0.37	4.22	2.36	1.69	0.43	0.67
Mobile apps	2.02	1,338	3,157	0.43	5.83	2.53	1.57	0.36	0.72
Cipher suites	2.02	1,337	7,827	0.44	7.49	1.78	1.56	0.57	0.62
Phone scam	1.90	1,256	6,729	0.38	5.28	2.57	1.61	0.30	0.74
Block cipher	1.88	1,242	3,923	0.43	5.43	1.95	1.55	0.67	0.57
DNS	1.86	1,232	3,701	0.37	4.07	2.18	1.66	0.49	0.64
Data backup	1.79	1,180	3,336	0.42	5.07	2.65	1.94	0.41	0.65
Credit card	1.77	1,169	4,361	0.40	5.80	2.32	1.84	0.42	0.66
Disk encryption	1.65	1,090	2,770	0.46	4.43	2.11	1.67	0.44	0.67
Virtual machine	1.53	1,013	3,024	0.50	6.00	2.01	1.55	0.46	0.67
SQL injection	1.47	972	4,248	0.37	3.20	2.25	1.56	0.44	0.68
Digital signature	1.22	807	2,219	0.41	3.91	2.13	1.70	0.52	0.62

**Figure 2:**
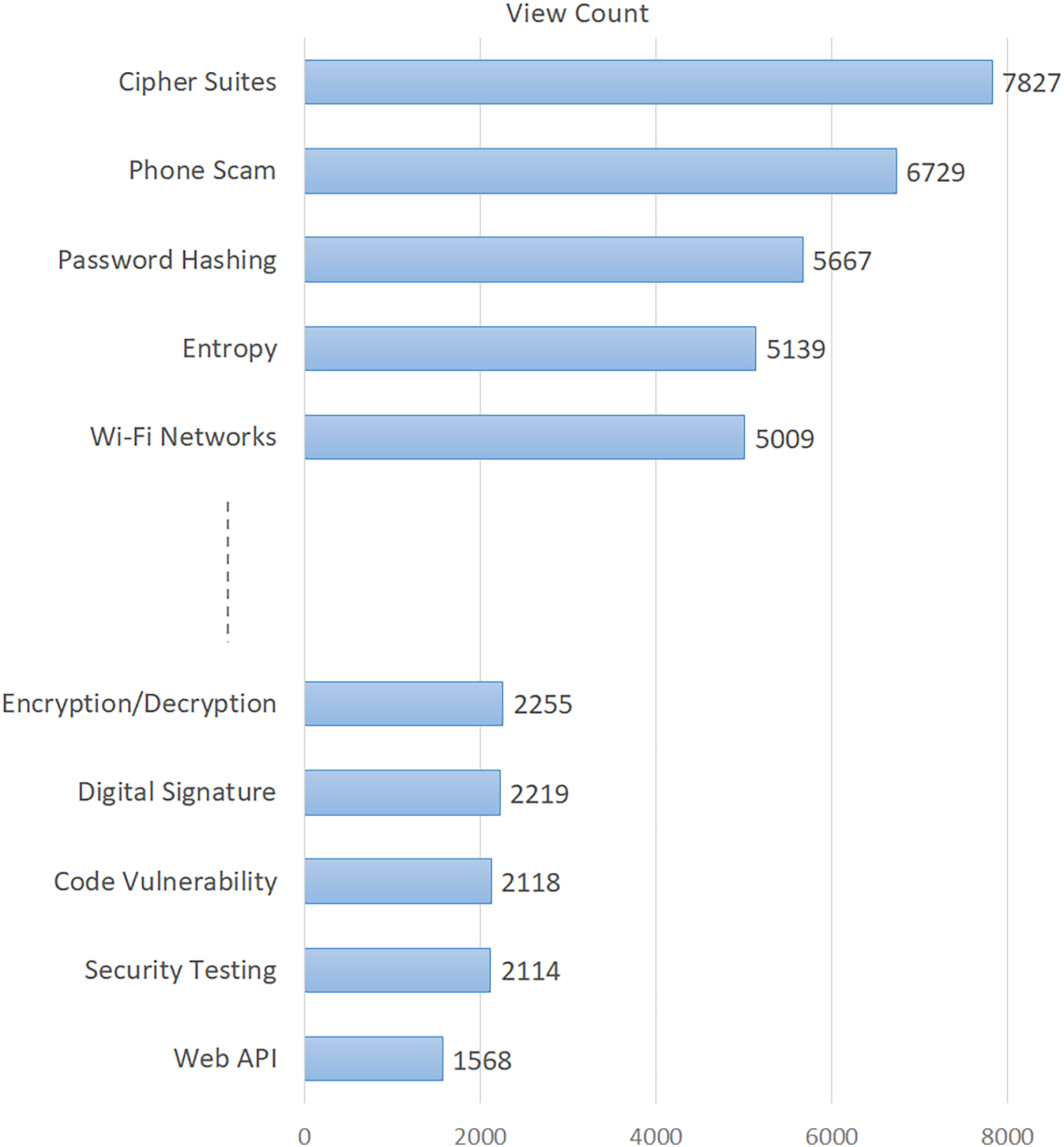
Average number of views of the topics.

**Figure 3:**
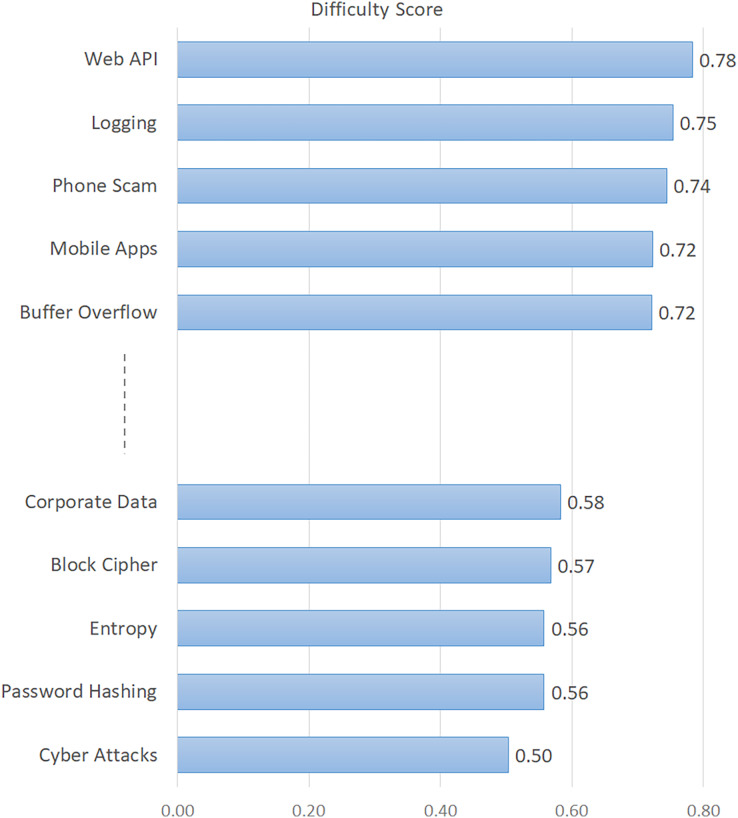
Difficulty levels of the topics.

The topics in this table are listed in descending order of their percentages. In addition, the difficulty score indicating the difficulty level of the topics is given in the last column of the table. The findings in Table 3 provide important metrics about various characteristics of information security issues. In order to give a visual sense, we also showed how popular the topics were in Fig. 2 and how difficult they were in Fig. 3. Based on Fig. 2, the five topics with the highest number of views are “Cipher Suites”, “Phone Scam”, “Password Hashing”, “Entropy”, and “Wi-Fi Networks”. Conversely, the topics with the lowest viewership include “Web API”, “Security Testing”, “Code Vulnerability”, “Digital Signature”, and “Encryption/Decryption”. Based on the difficulty score shown in Fig. 3, the topic with the highest level of difficulty is “Web API”, with a score of 0.78. The other most difficult topics are “Logging”, “Phone Scam”, “Mobile Apps”, and “Buffer Overflow”. On the other hand, “Cyber Attacks” and “Password Hashing” emerged as the most easily answered topics.

### In the field of information security, which tasks, techniques, and tools are the most frequently employed? (RQ4)

Information security entities, encompassing a diverse range of tasks, strategies, and technologies, are currently prevalent in all modern information ecosystems. Trends in information security technologies are constantly evolving simultaneously with technological transformations in information ecosystems. From this perspective, we have extended our investigation to find the linkages between information security challenges and information security-related technologies at this time. Every question post on the ISSE platform is accompanied by user-defined tags that describe the specific context and background of the question. The user selects these tags and includes them in their question. Tags are informative keywords that users assign to their inquiries to indicate the specific techniques, technologies, and tools related to information security. In order to get a particular set of tags correlated to information security, we initially segregated the tags of each post into separate entities and then computed the frequency of each tag over all postings. Consequently, we recognized a grand total of 1,257 unique tags that are frequently employed in the realm of information security. From these tags, we have determined the top 50 tags that appear most frequently. We have calculated their percentages and presented them in descending order in Table 4. According to Table 4, “encryption” is the most prominent tag, followed by “tls”, “authentication”, “passwords” and “web-application”.

**Table 4:** Top 50 information security-related tags.

No	Tag	Rate (%)	No	Tag	Rate (%)
1	Encryption	3.22	26	Exploit	0.72
2	Tls	3.18	27	Penetration-test	0.72
3	Authentication	2.47	28	Certificate-authority	0.70
4	Passwords	2.22	29	Openssl	0.69
5	Web-application	1.87	30	Attacks	0.69
6	Network	1.58	31	Ssh	0.66
7	Certificates	1.56	32	Firewalls	0.64
8	Malware	1.38	33	Mobile	0.59
9	Cryptography	1.31	34	Vulnerability	0.55
10	Hash	1.25	35	Virus	0.55
11	Windows	1.21	36	Appsec	0.54
12	Linux	1.16	37	Webserver	0.53
13	Privacy	1.09	38	Sql-injection	0.51
14	Email	0.98	39	Digital-signature	0.49
15	Web-browser	0.91	40	Aes	0.47
16	Php	0.90	41	Key-management	0.47
17	Xss	0.83	42	Cookies	0.45
18	Wifi	0.82	43	Password-cracking	0.43
19	Http	0.80	44	Brute-force	0.43
20	Android	0.75	45	Dns	0.43
21	Javascript	0.74	46	Gnupg	0.42
22	Vpn	0.74	47	Databases	0.41
23	Man-in-the-middle	0.74	48	Rsa	0.41
24	Public-key-infrastructure	0.73	49	Account-security	0.40
25	Password-management	0.72	50	Java	0.40

Furthermore, in order to offer insight into the time-related patterns of the top 50 tags, we displayed the yearly ratios, trend ratios, and orientations of the tags in Table 5. The general trend values of the tags are demonstrated in the last column of Table 5 and are sorted from increasing to decreasing. As can be seen in Table 5, the prominent tags with a rising trend are “malware”, “tls”, “certificates”, “openssl” and “account-security”. In contrast, the tags seeing a decline in popularity were identified as “appsec”, “web-application”, “network”, “cryptography”, and “penetration-test”.

**Table 5:** Time-based trends in the top 50 information security-related tags.

Tag	2010	2011	2012	2013	2014	2015	2016	2017	2018	2019	2020	2021	2022	Trend
Malware	0.19	1.06	1.15	1.22	1.39	1.35	1.35	1.52	1.41	1.42	1.54	1.54	1.51	0.10
Tls	1.63	2.89	3.36	3.52	4.28	4.04	3.09	3.07	2.57	2.50	2.76	2.59	2.90	0.10
Certificates	0.29	1.35	1.77	1.46	1.84	1.85	1.49	1.69	1.31	1.31	1.51	1.60	1.37	0.08
Openssl	0.00	0.02	0.26	0.61	0.99	0.86	0.76	0.81	0.60	0.73	0.67	0.53	0.80	0.06
Account-security	0.00	0.02	0.01	0.00	0.01	0.01	0.32	0.56	0.69	0.85	0.86	0.84	0.77	0.06
Man-in-the-middle	0.00	0.07	0.54	0.73	0.81	0.91	0.83	0.67	0.71	0.86	0.83	0.61	0.71	0.05
Vpn	0.10	0.46	0.54	0.66	0.60	0.75	0.78	0.71	0.83	0.78	1.05	0.74	0.80	0.05
Android	0.00	0.34	0.44	0.61	0.58	0.77	0.87	0.88	0.81	0.89	0.76	0.87	0.68	0.05
Vulnerability	0.10	0.02	0.00	0.01	0.45	0.63	0.73	0.67	0.79	0.80	0.56	0.59	0.76	0.05
Encryption	2.02	4.55	3.88	4.11	4.02	3.71	3.13	2.81	2.83	2.39	2.61	2.65	2.67	0.05
Privacy	0.29	0.72	1.18	1.17	1.09	1.18	1.20	1.17	0.98	1.01	0.95	1.17	0.93	0.05
Gnupg	0.00	0.02	0.05	0.18	0.44	0.55	0.47	0.51	0.45	0.47	0.49	0.49	0.63	0.05
Linux	0.58	1.28	1.29	1.23	1.37	1.08	1.17	1.11	1.27	1.00	1.12	0.91	1.10	0.04
Email	0.38	0.79	1.32	1.11	1.23	0.94	0.99	0.83	0.75	0.98	0.84	1.17	0.88	0.04
Wifi	0.19	1.11	0.84	0.78	0.77	0.71	0.94	1.10	0.72	0.82	0.76	0.58	0.68	0.04
Password-cracking	0.00	0.00	0.01	0.24	0.21	0.45	0.53	0.61	0.59	0.50	0.57	0.50	0.47	0.04
Certificate-authority	0.29	0.53	0.61	0.69	0.74	0.88	0.64	0.86	0.61	0.68	0.65	0.59	0.76	0.04
Javascript	0.38	0.74	0.60	0.91	0.71	0.71	0.76	0.63	0.76	0.80	0.79	0.75	0.82	0.03
Digital-signature	0.10	0.51	0.42	0.38	0.53	0.61	0.50	0.43	0.41	0.52	0.48	0.53	0.52	0.03
Ssh	0.38	0.50	0.69	0.76	0.80	0.73	0.66	0.65	0.60	0.51	0.59	0.58	0.79	0.03
Public-key-infrastructure	0.19	0.87	1.03	0.83	1.11	0.95	0.68	0.65	0.52	0.52	0.62	0.50	0.58	0.03
Hash	0.58	1.56	1.86	1.44	1.72	1.35	1.11	1.10	0.98	1.03	1.08	1.13	0.95	0.03
Windows	0.96	1.73	1.90	1.45	1.36	1.18	1.08	1.13	1.00	0.94	1.10	1.19	1.26	0.02
Rsa	0.00	0.02	0.01	0.41	0.62	0.63	0.42	0.39	0.38	0.44	0.39	0.38	0.30	0.02
Virus	0.29	0.60	0.75	0.55	0.49	0.65	0.52	0.55	0.54	0.44	0.62	0.43	0.54	0.02
Aes	0.00	0.07	0.46	0.72	0.59	0.62	0.49	0.38	0.50	0.36	0.37	0.45	0.22	0.02
Xss	0.58	0.60	0.64	0.91	0.90	0.66	0.83	0.75	0.93	1.02	1.01	0.78	0.79	0.02
Http	0.67	0.50	0.79	0.88	0.78	0.80	0.76	0.81	0.76	0.81	0.91	0.89	0.88	0.02
Brute-force	0.19	0.70	0.45	0.51	0.44	0.41	0.38	0.40	0.50	0.38	0.40	0.39	0.33	0.01
Sql-injection	0.38	0.39	0.53	0.68	0.52	0.51	0.54	0.45	0.50	0.52	0.52	0.38	0.52	0.01
Dns	0.48	0.43	0.46	0.36	0.31	0.44	0.51	0.38	0.50	0.35	0.46	0.44	0.49	0.00
Cookies	0.58	0.34	0.53	0.41	0.53	0.35	0.34	0.42	0.47	0.48	0.57	0.52	0.52	0.00
Password-management	0.38	1.04	0.89	0.82	0.78	0.80	0.77	0.70	0.71	0.63	0.60	0.52	0.32	−0.01
Java	0.38	0.39	0.57	0.59	0.48	0.54	0.38	0.33	0.34	0.26	0.30	0.26	0.25	−0.01
Key-management	0.58	0.92	0.49	0.59	0.66	0.53	0.44	0.40	0.43	0.29	0.37	0.33	0.30	−0.02
Authentication	2.50	3.18	2.74	3.08	2.84	2.49	2.45	2.05	2.32	2.31	2.16	2.32	2.21	−0.02
Databases	0.67	0.56	0.55	0.41	0.51	0.45	0.46	0.40	0.33	0.30	0.37	0.32	0.28	−0.03
Exploit	1.06	0.56	0.90	0.88	0.80	0.74	0.79	0.81	0.64	0.60	0.43	0.61	0.65	−0.03
Web-browser	1.25	1.45	1.27	1.26	1.02	0.86	0.86	0.74	0.82	0.74	0.73	0.85	0.80	−0.03
Mobile	0.87	1.18	0.82	0.71	0.55	0.70	0.64	0.49	0.55	0.49	0.43	0.35	0.41	−0.04
Firewalls	1.25	1.03	1.17	0.92	0.65	0.68	0.57	0.60	0.47	0.49	0.51	0.32	0.65	−0.05
Passwords	2.31	3.08	3.34	2.84	2.83	2.21	2.02	1.89	1.89	2.02	1.89	1.70	1.48	−0.06
Webserver	1.35	1.01	0.84	0.77	0.65	0.70	0.42	0.40	0.44	0.38	0.33	0.30	0.35	−0.08
Php	1.54	0.77	1.19	1.21	1.23	0.93	0.88	0.83	0.92	0.80	0.63	0.62	0.43	−0.09
Attacks	1.83	1.37	1.22	0.72	0.85	0.83	0.61	0.65	0.53	0.50	0.43	0.52	0.36	−0.11
Penetration-test	2.50	0.91	1.15	0.95	0.77	0.71	0.83	0.64	0.71	0.69	0.37	0.29	0.30	−0.17
Cryptography	3.08	5.60	2.26	1.63	1.44	1.46	1.02	0.96	0.92	0.87	0.81	0.54	0.76	−0.18
Network	4.90	3.54	2.65	1.86	1.58	1.48	1.60	1.52	1.33	1.07	1.21	0.95	1.34	−0.27
Web-application	5.96	3.30	2.95	2.89	1.93	1.52	1.70	1.44	1.71	1.81	1.70	1.18	1.14	−0.37
Appsec	7.69	3.52	1.11	0.74	0.40	0.35	0.37	0.27	0.34	0.30	0.24	0.23	0.19	−0.58

In the concluding phase of our investigation, we have determined the top 15 tags that appear most frequently for each of the 38 topics. These findings are presented in Table A2, where the topics are arranged in descending order based on their respective percentages. Similarly, these tags in each row are sorted for each topic in descending order. In this sense, Table A2 provides an understanding of which information security issues are related to which tags and how much. For example, for the “Cyber Attacks” topic in the first row of the table, the “encryption” tag has the highest frequency, while the “denial-of-service” tag has the lowest frequency. This categorization of the tags, given in Table A2, reveals both the nature and scope of the issues and the tools and technologies with which they are related. Therefore, categorizing tags under specific problems significantly contributes to a more accurate understanding of the problems and their rapid and effective resolution. It also informs users about the origin and background of the questions, as well as related tasks, technologies, and experiences.

## Discussion

Our research carried out an in-depth analysis to identify information security-oriented issues and their descriptive dimensions. Our study has revealed important findings for understanding the current issues in the field of information security, interactions and discussions based on information security, workflows, security paradigms, and solution proposals. We now discuss these findings in detail. As a result of our analysis, information security issues were identified, represented by 38 different topics discovered by LDA. In order to make the main themes of information security problems more understandable, 38 information security topics are classified under seven basic categories: “System Security” (24.25%), “Cryptography” (20.85%), “Network Security” (18.02%), “Web Security” (14.64%), “Data Security” (10.76%), “Software Security” (7.56%), and “Mobile Security” (3.93%) (see Fig. 1). Although there are seven categories, the topics in the “System Security” (24.25%) and “Cryptography” (20.85%) categories constitute about half of the information security issues. Therefore, we can say that the issues in the “System Security” and “Cryptography” categories are the dominant problems for information security (Whitman & Mattord, 2021). Among the 38 issues discovered as a topic, the top five are “Cyber Attacks”, “Security Testing”, “Certification”, “User Account”, and “Wi-Fi Networks”. Today, even highly secure systems can be exposed to various types of cyber-attacks. Preventing these cyber-attacks and making information secure against them are among the primary problems that need to be solved in information security (Ashibani & Mahmoud, 2017). The fact that “Cyber Attacks” and “Security Testing” are in the first two places among security problems is a finding that confirms this inference (Stiawan et al., 2017).

Furthermore, we conducted an examination of the chronological patterns of information security issues and derived several significant insights regarding their tendencies. “Certification”, “Encryption Keys”, “Web API”, “Disk Encryption”, and “VPN” are the ones with the most increasing trend. On the other hand, “Security Testing”, “Cyber Attacks”, and “Corporate Data” emerged as the issues with the most decreasing trend. It is noteworthy that although “Cyber Attacks” and “Security Testing” are the two topics with the highest percentage, they have the most decreasing trend. We can explain this finding by assuming that more questions are asked about new technologies. When interpreting the most increasing or decreasing trends about the issues, it is necessary to take into account that some of the main issues related to information security have reached saturation (Yang et al., 2016). Indeed, there is a reduced likelihood of fresh inquiries being posed regarding older topics. The reason for not addressing these issues is not due to their decreased significance, but rather because numerous questions regarding these topics have previously been posed and resolved (Croft et al., 2022). Questions asked on Q&A platforms such as ISSE or Stack Overflow are not allowed to be asked again. Since questions about old problems have been asked before, new questions are mostly asked about new technologies.

Currently, there has been an increase in questions regarding information security issues related to emerging technologies. This is mostly due to the novelty of these topics and the fact that many of the problems surrounding them have not yet been addressed (Ammar, Russello & Crispo, 2018; Lopez et al., 2019). In this respect, it has been observed that more questions have been asked in recent years on topics such as “Certification” and “Encryption Keys”, which are relatively up-to-date and place more emphasis on the security of sensitive personal information (Ammar, Russello & Crispo, 2018). Another finding that confirms this inference is that although issues such as “Cyber Attacks”, “Security Testing”, “User Account”, “XSS Attacks”, “Corporate Data”, “Port Scanning”, and “SSH Access” are in the top ranks, most of them have a decreasing trend (see Tables 1 and 3).

To gain a deeper understanding of the critical, widely discussed, and challenging concerns in information security, we conducted an analysis of several aspects of these topics. We considered certain descriptive indicators of the posts, which allowed us to broaden our conclusions. The indicators for each question on the ISSE platform offer a diverse range of understandings related to the topics investigated (Croft et al., 2022). A question would not be asked again if it has previously been asked. Alternatively, by consulting the earlier query and its related responses, the user may discover the answer (Lopez et al., 2018; Uddin et al., 2021). The total number of views pertaining to the issues is thus a significant metric for determining their level of popularity. In this context, “Cipher Suites”, “Phone Scam”, “Password Hashing”, “Entropy”, and “Wi-Fi Networks” emerged as the most popular (most viewed) topics (see Fig. 2). According to the difficulty scores, the most difficult issues are “Web API”, “Logging”, “Phone Scam”, “Mobile Apps”, and “Buffer Overflow” (see Fig. 3). The findings in this context emphasized that such issues that have not yet been resolved in the field of information security require more consideration and effort towards a solution (Yang et al., 2016).

Furthermore, our analysis yielded significant findings indicating a substantial correlation between information security challenges and cybersecurity technologies that encompasses a diverse set of activities, methodologies, and capabilities. According to our findings, “encryption” is the most used tag in questions, followed by “tls”, “authentication”, “passwords” and “web-application”. These tags, which are most commonly used in information security questions, clearly emphasize the importance of securely transferring sensitive information about users, such as user credentials, accounts, credit cards, payment details, or login details (McCormac et al., 2017; Ammar, Russello & Crispo, 2018). Our analysis, which takes into account the temporal trends of the tags, revealed “malware”, “tls”, “certificates”, “openssl”, and “account-security” as the top five tags with an increasing trend. The emergence of “malware” in the first place is an important finding that reveals that malicious software variants are seen as among the most important threats to information security in the near future (Abraham & Chengalur-Smith, 2010). These top five tags (“malware”, “tls”, “certificates”, “openssl”, and “account-security”) with the most increasing trend, also highlight technologies for the protection and secure transmission of sensitive information such as user accounts and personal confidential data (see Table 5).

Finally, we identified which cybersecurity tags are used to ask about which information security issues. In this way, we showed the top 15 most prominent tags for each of the 38 topics (see Table A2). For example, “encryption”, “authentication”, “tls”, “passwords”, and “web-application” emerged as the most used tags in the questions about the “Cyber Attacks” topic in the first row of Table A2. The findings of this analysis provided important insights into which technical tags are used to describe information security questions and issues (Yang et al., 2016; Croft et al., 2022).

### Implications for researchers and practitioners

Information security covers the measures and activities necessary to ensure the safety of users, the safety of the data, and the protection from malicious activities. With the effect of technological developments and the increase in malicious activities, it is more important than ever to be conscious of information security and take the necessary safety measures (Abraham & Chengalur-Smith, 2010). The issues and user experiences shared on ISSE and similar Q&A platforms can be considered an important source of information and motivation for efforts to solve information security issues (Croft et al., 2022). The experimental backdrop, methods, and outcomes of this investigation may have noteworthy ramifications and instructions that will contribute to the understanding of domain-specific issues for information security communities and enable them to take the necessary security measures (McCormac et al., 2017). Our findings may guide us in taking the necessary measures to protect data, ensure the safety of computer networks, and identify and implement security policies (Furnell, Fischer & Finch, 2017). Our research is aimed at assisting a wide range of information security stakeholders, including practitioners, instructors, academics, developers, and enthusiasts.

Developers may drive the evolution of the area of information security by developing more particular apps and devices to solve contemporary information security concerns and demands, as indicated in our outcomes. Tool developers can create useful libraries or tools for popular and difficult issues. Inferences to be drawn from the issues expressed on online sharing platforms such as ISSE can help the research community better understand the issues and difficulties encountered in information security (Croft et al., 2022). Although all of the concerns highlighted are relevant in and of themselves, the results we obtain suggest that information security researchers should focus on the most prominent and problematic challenges. For example, the topics in the “System Security” and “Cryptography” categories are about half of the information security issues. In particular, the issues in these two categories can be considered priorities, and faster solutions can be produced (Silic & Back, 2014). Furthermore, researchers can take advantage of our research methodology to discuss different contexts of information security and thus expand our analysis.

Information security instructors can organize online training programs and webinars to emphasize the importance of being conscious of information security and taking the necessary security measures. In particular, considering the most popular and most challenging security problems, a more effective training curriculum can be prepared for information security trainings (Furnell, Fischer & Finch, 2017). Instructors may maintain their educational programs and curriculum up to date with current developments and give a modern understanding of information security potential hazards (Glaspie & Karwowski, 2018). Our analysis may be utilized by ISSE or other Q&A services to define, label, and better categorize user posts within a systematic taxonomy (Barua, Thomas & Hassan, 2014). Our consequences may be helpful to readers in general and information security enthusiasts to stay updated on the latest advancements and trends in the area.

## Conclusions

This study seeks to shed light on prevalent issues and difficulties surrounding information security. Considering the lack of experimental research in this field, we analyzed all posts shared on ISSE, a Q&A platform specific to information security, using a semi-automatic methodology based on the LDA topic modeling approach. In addition, we have found what the most commonly used cyber security technologies are and what issues they are related to. As a result of this analysis, 38 topics presenting the current landscape of information security issues and trends were discovered and classified into seven categories. Within these categories, approximately half of the information security issues are topics in the categories “System Security” and “Cryptography”. Among the 38 issues discovered as a topic, the most prominent issues are “Cyber Attacks”, “Security Testing”, and “Certification”. On the other hand, “Certification”, “Encryption Keys”, and “Web API” have emerged as the topics with the most increasing trends. In addition, we also investigated the popularity and difficulty metrics of the issues by addressing different dimensions of them. As a result, we found that “Cipher Suites”, “Phone Scam”, and “Password Hashing” were the most popular (most viewed) issues. Considering the difficulty score of the issues, we identified “Web API”, “Logging”, and “Phone Scam” as the most difficult issues. Our investigation also indicated that information security challenges are inextricably associated with cyber security technologies (tasks, practices, and tools). In this context, “encryption”, “tls”, “authentication”, “passwords”, and “web-application” came to the fore as the most frequently used tags in questions.

Our study opens doors to information security stakeholders in different profiles to improve information security architecture, techniques, and tools. Developers can provide better support tools and documentation by taking advantage of our findings. Information security experts and educators can plan curriculum and training by taking these findings into consideration. Researchers can direct their focal points toward popular and difficult issues and propose more solutions for these problems. We envisage that more research should be carried out in order to ensure continuous follow-up on information security issues, experiences, and awareness in the field. In the near future, we intend to expand our study to focus on the subcategories of information security and conduct research that focuses on the most difficult and popular issues.

## Supplemental Information

10.7717/peerj-cs.1954/supp-1 Supplemental Information 1 Source Codes.

10.7717/peerj-cs.1954/supp-2 Supplemental Information 2 Details about the topics generated by LDA.

10.7717/peerj-cs.1954/supp-3 Supplemental Information 3 Topics and related tags (tasks, techniques, and tools).

## Additional Information and Declarations

### Data Availability

The following information was supplied regarding data availability: The data is available at figshare: Gurcan, Fatih (2024). Information Security Stack Exchange Data. figshare. Dataset. https://doi.org/10.6084/m9.figshare.25358080.v1.
