# Supplementary material for: What are developers talking about information security? A large-scale study using semantic analysis of Q&A posts
Source: PeerJ Comput Sci. 2024 Mar 26;10:e1954. doi: 10.7717/peerj-cs.1954 (PMC11041951; doi:10.7717/peerj-cs.1954)
Supplement: Supplemental Information 2 [file peerj-cs-10-1954-s002.docx]

**Table A1.** Details about the topics generated by LDA

| Topic Name | Descriptive LDA keywords | % |
| --- | --- | --- |
| Cyber Attacks | attack security attacker against system time risk secure case protect problem point consider take compromise prevent people reason probably issue | 5.75 |
| Security Testing | security tool test system policy information level find standard risk management requirement control software analysis learn team process penetration base | 3.93 |
| Certification | certificate trust cert root sign ssl issue authority chain cas name browser valid check crl intermediate create self-signed csr store | 3.90 |
| User Account | password user account login username change store log access security enter reset manager secure site credentials system database attacker website | 3.82 |
| Wi-Fi Networks | network router wifi connect device mac access traffic packet internet home computer wireless attack address connection arp sniff ap point | 3.69 |
| XSS Attacks | code script xss javascript php html input attack execute page tag string vulnerability url character form data function exploit inject | 3.66 |
| Corporate Data | company information data people customer personal service access person business employee privacy give security government law legal take country case | 3.65 |
| Logging | try find time log change check problem show start help error run happen attempt tell issue give follow test take | 3.61 |
| Website | site browser website page google user chrome link url web firefox visit click search extension redirect information open content access | 3.40 |
| Port Scanning | port firewall packet scan ip block traffic rule tcp network connection attack open nmap service host run udp ddo filter | 3.14 |
| SSH Access | server access ssh host machine service web network run remote connect security local internal secure internet cloud allow connection aws | 3.00 |
| Encryption Keys | key private public generate secret rsa encrypt pair symmetric share create decrypt master gpg passphrase pgp hsm message asymmetric sign | 2.96 |
| Malware | windows malware computer software install virus run machine malicious program detect system pc infect antivirus download scan microsoft infected av | 2.95 |
| Access Control | user access run system root permission windows admin command privilege process directory account allow group file administrator set shell script | 2.74 |
| Password Hashing | hash password salt function hashing algorithm store value bcrypt crack table attacker collision database generate find time string compare input | 2.70 |
| Web API | application user app web api service access data secure store security server call database solution client request code rest approach | 2.68 |
| TLS Connection | server client send tls connection ssl protocol authentication handshake side message communication secure session http connect attacker mitm exchange attack | 2.63 |
| CSRF | token session cookie user request csrf jwt store send server access log refresh login value set attacker form authentication browser | 2.53 |
| Code Vulnerability | vulnerability code version exploit source update find bug software vulnerable security patch package fix java issue report release open library | 2.42 |
| Entropy | number character random generate entropy bit time word length force long brute take letter guess crack dictionary digit passphrase try | 2.38 |
| HTTP Proxy | http request header proxy response send url server browser redirect traffic post web attack hst site ssl set connection burp | 2.37 |
| E-Mail | email send address mail account link spam e-mail gmail receive message user smtp sender google spf phishing recipient service contact | 2.23 |
| Buffer Overflow | code function program address buffer return stack exploit overflow memory payload write execute call shell metasploit shellcode binary reverse try | 2.19 |
| Authentication | authentication user token code access authorization authenticate service oauth credentials identity factor provider secret flow auth resource google otp login | 2.16 |
| VPN | vpn tor traffic network node proxy ip isp connection internet connect tunnel service hide openvpn provider ipsec address route anonymous | 2.15 |
| Encryption/Decryption | encrypt data encryption key decrypt store secure decryption text information sensitive protect access storage plaintext plain unencrypted send system method | 2.14 |
| File Transmission | file image upload download pdf document folder contain format open directory create content text copy save name zip type tool | 2.09 |
| Mobile Apps | device android app phone mobile ios apple access iphone fingerprint camera lock keyboard hardware security install pin bluetooth smartphone screen | 2.02 |
| Cipher Suites | cipher algorithm support rsa openssl tls suite encryption exchange implementation protocol dh version key curve prime bit cryptography parameter standard | 2.02 |
| Phone Scam | phone number message call facebook send mobile sms account video whatsapp location google chat sim friend receive signal text service | 1.90 |
| Block Cipher | byte value block message bit size mode string length encode output data mac cipher k random algorithm number result format | 1.88 |
| DNS | ip address domain dns name server record host spoof subdomain request block different isp cloudflare list query hostname website find | 1.86 |
| Data Backup | drive data usb backup disk delete hard file write computer copy flash wipe storage recover memory erase ssd space stick | 1.79 |
| Credit Card | card bank credit pcus pin payment number transaction data store account information smart customer dss detail compliance online requirement compliant | 1.77 |
| Disk Encryption | boot disk tpm system partition laptop drive hardware handshake firmware volume computer os bitlocker truecrypt secure encryption bio device veracrypt | 1.65 |
| Virtual Machine | linux machine system run vm memory os kernel virtual process host window hardware cpu kali sandbox access guest driver code | 1.53 |
| SQL Injection | database sql injection query table mysql db string parameter data error php statement column code vote result sqlmap return try | 1.47 |
| Digital Signature | signature sign message verify bob alice trust digital integrity send party verification prove check hash provide document signing time tamper | 1.22 |
