# Supplementary material for: What are developers talking about information security? A large-scale study using semantic analysis of Q&A posts
Source: PeerJ Comput Sci. 2024 Mar 26;10:e1954. doi: 10.7717/peerj-cs.1954 (PMC11041951; doi:10.7717/peerj-cs.1954)
Supplement: Supplemental Information 3 [file peerj-cs-10-1954-s003.docx]

**Table A2.** Topics and related tags (tasks, techniques, and tools)

| Topic Name | Related Tags |
| --- | --- |
| Cyber Attacks | encryption authentication tls passwords web-application network attacks cryptography hash web-browser linux man-in-the-middle http ddos denial-of-service |
| Security Testing | penetration-test professional-education web-application audit appsec network risk-management career encryption terminology certification corporate-policy compliance authentication threat-modeling |
| Certification | certificates tls certificate-authority public-key-infrastructure openssl x.509 man-in-the-middle certificate-revocation authentication digital-signature windows encryption web-browser cryptography ocsp |
| User Account | passwords password-management authentication password-policy encryption web-application hash account-security multi-factor brute-force email password-cracking password-reset windows web-browser |
| Wi-Fi Networks | wifi network wireless router man-in-the-middle wpa2 arp-spoofing mac-address wireshark aircrack-ng encryption sniffer tls vpn attacks |
| XSS Attacks | xss javascript web-application php html injection web-browser appsec content-security-policy exploit penetration-test vulnerability encoding reflected-xss sql-injection |
| Corporate Data | privacy legal encryption tls email authentication passwords web-application data-leakage network physical vpn anonymity hipaa disclosure |
| Logging | malware linux php encryption tls windows network attacks authentication web-application webserver logging apache virus passwords |
| Website | web-browser tls privacy chrome web-application malware javascript firefox google phishing http cookies browser-extensions url-redirection authentication |
| Port Scanning | firewalls network nmap ports ids network-scanners ddos snort tcp denial-of-service attacks linux iptables windows penetration-test |
| SSH Access | ssh network webserver tls firewalls linux server web-application vpn encryption windows authentication apache aws cloud-computing |
| Encryption Keys | encryption gnupg pgp public-key-infrastructure key-management cryptography rsa ssh tls certificates asymmetric digital-signature openssl authentication key-generation |
| Malware | malware virus antivirus windows antimalware ransomware network linux windows-10 trojan exploit keyloggers macos virus-removal web-browser |
| Access Control | linux windows privilege-escalation access-control permissions docker authentication active-directory passwords ssh sudo unix php network bash |
| Password Hashing | hash passwords salt password-cracking cryptography encryption bcrypt authentication md5 sha password-management brute-force pbkdf2 php john-the-ripper |
| Web API | authentication web-application api encryption rest mobile tls android web-service appsec oauth databases passwords javascript java |
| TLS Connection | tls authentication encryption man-in-the-middle certificates openssl cryptography http network ssh web-application passwords public-key-infrastructure protocols key-exchange |
| CSRF | csrf cookies authentication web-application session-management jwt xss token php oauth javascript http web-browser oauth2 tls |
| Code Vulnerability | vulnerability cve exploit known-vulnerabilities linux java vulnerability-scanners web-application opensource source-code openssl tls penetration-test windows heartbleed |
| Entropy | passwords random brute-force password-cracking entropy encryption cryptography hash password-policy authentication passphrase password-management key-generation aes dictionary |
| HTTP Proxy | tls http proxy web-application man-in-the-middle burp-suite hsts cors web-browser csrf header webserver http-proxy authentication apache |
| E-Mail | email spam email-spoofing phishing gmail smtp authentication encryption privacy spf account-security dkim dmarc passwords tls |
| Buffer Overflow | buffer-overflow exploit metasploit c shellcode exploit-development linux windows malware memory aslr reverse-engineering assembly meterpreter penetration-test |
| Authentication | authentication oauth multi-factor oauth2 openid-connect authorization saml one-time-password sso kerberos passwords openid web-application google totp |
| VPN | vpn tor privacy anonymity network proxy tls encryption ipsec openvpn ip man-in-the-middle firewalls isp wifi |
| Encryption/Decryption | encryption cryptography aes key-management file-encryption databases decryption passwords tls disk-encryption php web-application password-management hash authentication |
| File Transmission | malware file-upload encryption php pdf virus web-application file-encryption windows steganography zip linux exploit image passwords |
| Mobile Apps | android mobile ios encryption bluetooth privacy iphone authentication malware hardware smartphone physical usb biometrics passwords |
| Cipher Suites | tls encryption cryptography openssl rsa cipher-selection diffie-hellman ciphers ecc certificates aes ssh public-key-infrastructure fips key-exchange |
| Phone Scam | phone mobile privacy sms encryption android facebook whatsapp authentication simcard smartphone gsm spoofing multi-factor network |
| Block Cipher | encryption cryptography aes hash tls hmac openssl authentication rsa digital-signature encoding passwords certificates decryption file-encryption |
| DNS | dns ip network tls dnssec dns-domain ip-spoofing dns-spoofing certificates domain ddos malware privacy web-application firewalls |
| Data Backup | deletion forensics encryption usb-drive disk-encryption data-recovery hardware malware windows usb backup ssd linux storage data-leakage |
| Credit Card | pci-dss credit-card banks smartcard encryption authentication rfid pci-scope compliance nfc fraud payment-gateway tls emv web-application |
| Disk Encryption | encryption disk-encryption tpm linux bitlocker windows hardware bios luks boot trusted-computing truecrypt malware veracrypt firmware |
| Virtual Machine | virtualization linux malware windows operating-systems memory hardware meltdown spectre sandbox kernel encryption exploit kali-linux network |
| SQL Injection | sql-injection mysql php web-application databases sqlmap penetration-test injection sql-server hydra encryption appsec passwords vulnerability authentication |
| Digital Signature | digital-signature encryption authentication cryptography pgp public-key-infrastructure gnupg hash certificates integrity tls timestamp certificate-authority man-in-the-middle code-signing |
